# The GBD Brazil network: better information for health policy decision-making in Brazil

**DOI:** 10.1186/s12963-020-00224-1

**Published:** 2020-09-30

**Authors:** Deborah Carvalho Malta, Valéria Maria de Azeredo Passos, Ísis Eloah Machado, Maria de Fatima Marinho Souza, Antonio Luiz P. Ribeiro

**Affiliations:** 1 Escola de Enfermagem, Universidade Federal de Minas Gerais, Belo Horizonte, MG Brazil; 2 Programa de Pós Graduação em Ciências da Saúde, Faculdade Ciências Médicas de Minas Gerais, Belo Horizonte, MG Brazil; 3 Escola de Medicina Universidade Federal de Outro Preto, Ouro Preto, Brazil; 4 Programa de Pós Graduação em Saúde Pública, Faculdade de Medicina, Universidade Federal de Minas Gerais, Belo Horizonte, Brazil; 5 Hospital das Clínicas and School of Medicine, Universidade Federal de Minas Gerais, Av. Alfredo Balena, 110, 1o andar, Belo Horizonte, MG 30130-100 Brazil

The Global Burden of Disease (GBD) project provides a long-term, comprehensive, and comparable picture of what disables and kills people across countries, time, age, and sex [1]. The GBD initiative begun in 1991 and the first results were published by the World Bank in the World Development Report 1993, with the full results of the GBD 1990 study published in the Lancet in 1997 [1]. Since then, the GBD study has grown in scope, relevance, participation, and scale, with several cycles of estimates and thousands of publications using data and metrics generated. Its publications are now among the most cited studies in the scientific literature and a main source for global health information.

The main goal of the GBD project is to provide comprehensive data on disease burden to support evidence-based decision-making and policies at local, regional, national, and global levels. Insights at the national and sub-national levels can be gained from the GBD estimates if provided with sufficient detail. For Brazil, considering the plurality of the society and its diversity across the regions of the country, obtaining sufficiently fine-grained burden estimates is challenging but needed.

Recognizing this challenge, the GBD Brazil Network was created in 2014 as a collaboration among the Brazilian Ministry of Health, a network of Universities, Research Institutes and Health Departments, led by the Universidade Federal de Minas Gerais (UFMG), and the Institute for Health Metrics and Evaluation (IHME) of the University of Washington, which leads the global project [2]. Currently, the GBD Brazil Network includes about 300 Brazilian collaborators, including scholars, researchers, health managers, and technicians from the Ministry of Health and the Health Departments. The aims of this Network are to provide methodological support and evaluate the estimates of the GBD study at national and subnational level, to evaluate and refine the GBD models, to produce evidence using GBD estimates, and to disseminate the culture of using data in decision-making processes related to health policy.

The GBD Brazil Network conducted several initiatives, both in academia and in health services, to discuss Brazilian health issues and to disseminate tools and metrics developed by the GBD project. These capacity-building and dissemination activities involved face-to-face training and postgraduate courses, lectures, media releases, scientific workshops, and regular meetings of a scientific committee, with participation of researchers from several different Brazilian Institutions and ad hoc participation of researchers from the IHME. A web course in the Portuguese language on GBD methods is being developed to be launched later in 2020.

Regarding the scientific production using GBD estimates, the GBD Brazil Network produced analysis that have resulted in the publication of two capstone papers [3, 4]. In 2017, the first set of articles from this group of researchers was published in a supplement to the *Brazilian Journal of Epidemiology* [2]. This second set of articles that is now being published in *Population Health Metrics* aims to further discuss and disseminate findings on the burden of disease in Brazil to the international academic and scientific community.

In the current supplement, we used the GBD tools and metrics developed by to explore different health conditions and specific populations in Brazil and its federative units, from 1990 to 2017. These studies describe patterns and trends, as well as knowledge gaps and point to possibilities for improving the models and estimates used by the GBD study.

The publication covers the life cycle of the Brazilian population, including an evaluation of inequalities in infant mortality and the burden of disease among older people. An emphasis was given to studies on chronic noncommunicable diseases, considering the recent epidemiological transition and including analyses on overall mortality from chronic diseases, and the disease burden of diabetes, hypertension, and breast cancer. Given the triple burden of disease, which continues to challenge the Brazilian health system, communicable diseases were not forgotten, with articles on tuberculosis and malaria. Violence by firearms, one of the main causes of death in the country, is also covered. There is also a comprehensive discussion on the impact of an integrated approach to risk factors for chronic noncommunicable diseases (obesity, physical activity, and smoking).

The GBD Brazil network is continuously discussing with IHME, researchers, and analysts the accuracy and reliability of the estimates, and it also monitors data quality in the country. An article on vital statistics data and another on the quality of death certificate completion calls attention to the advances and challenges concerning vital statistics in Brazil. Finally, the article on health-related “Sustainable Development Goals (SDGs)” analyzes the indicators and trends of 41 health-related SDG indicators in Brazil and its States and covers regional inequalities, aiming to leave no one behind.

Inequality and inequity have been hallmarks of Brazilian society; access to health care and basic sanitary services varies according to the socio-economical position, race/skin color, and region. The creation, 30 years ago, of the Brazilian Unified Health System (SUS—from Portuguese *Sistema Único de Saúde*) and its expansion in the last three decades has allowed Brazil to rapidly address the changing population health needs, with dramatic upscaling of health service coverage. However, the situation has deteriorated in the last 4 years of economic crisis and political instability, especially due to the reduction of investments and expenditures in health. Indeed, fiscal policies implemented in 2016 ushered in austerity measures that, alongside the new environmental, educational, and health policies of the Brazilian government, could reverse the hard-earned achievements of the SUS and threaten its sustainability and ability to fulfil its constitutional mandate of providing health care for all [5]. At present, the consequences of the COVID-19 pandemic are worsened by the health policies of the Brazilian government [6], leading to unpredictable and worrisome consequences for the health status of the Brazilian population, the health care system, and the Brazilian economy.

Maintaining and expanding the GBD Brazil Network will assure that the impact of these changes is estimated and the burden estimates remain available for Brazil to guide decisions and health policies in the future. The GBD Brazil Network intends to use GBD data to provide decision-makers at the local, regional, and national levels with the best and most up-to-date evidence on trends and drivers of population health to support decisions and health policy. Providing sound scientific evidence for local managers is challenging due to data gaps, high variability, and the low quality of data from small municipalities. The importance of using models and assumptions for small numbers must be considered as well.

Independent groups of researchers are vital in the analysis of country data, reflecting on current and future challenges, tracing trends, and analyzing the living and health conditions of the Brazilian population, from different perspectives. We are aware of the heterogeneity of the quantity and quality of secondary data among the different health conditions we address. Further research is needed, and we advocate for more resources, in Brazil, not only for health care, but also for research. Knowing the health status pattern and its trends in the last decades allows us to take a critical look at progress and what limits it in addressing disease burden. Improved health policies and a strengthened SUS are needed to build a fairer and more equitable society.

## Abbreviations

GBD: Global Burden of Disease
SUS: Brazilian Unified Health System (from Portuguese *Sistema Único de Saúde*)
SDGs: Sustainable development goals
UFMG: Universidade Federal de Minas Gerais
*IHME*: Institute for Health Metrics and Evaluation
